# Fe_2_O_3_ Embedded in N-Doped Porous Carbon Derived from Hemin Loaded on Active Carbon for Supercapacitors

**DOI:** 10.3390/molecules29010146

**Published:** 2023-12-26

**Authors:** Zitao Yang, Cunhao Luo, Ning Wang, Junshao Liu, Menglong Zhang, Jing Xu, Yongnan Zhao

**Affiliations:** 1 Fujian Provincial Key Laboratory of Eco-Industrial Green Technology, College of Ecology and Resources Engineering, Wuyi University, Wuyishan 354300, China; tzy1962@126.com (Z.Y.); lch1802023@126.com (C.L.);; 2 Tianjin Key Laboratory of Advanced Fiber and Energy Storage Technology, School of Material Science and Engineering, Tiangong University, Tianjin 300387, China

**Keywords:** Hemin/AC, Fe_2_O_3_/N-PC, pseudocapacitance, quasi-solid-state supercapacitor

## Abstract

The high power density and long cyclic stability of N-doped carbon make it an attractive material for supercapacitor electrodes. Nevertheless, its low energy density limits its practical application. To solve the above issues, Fe_2_O_3_ embedded in N-doped porous carbon (Fe_2_O_3_/N-PC) was designed by pyrolyzing Hemin/activated carbon (Hemin/AC) composites. A porous structure allows rapid diffusion of electrons and ions during charge–discharge due to its large surface area and conductive channels. The redox reactions of Fe_2_O_3_ particles and N heteroatoms contribute to pseudocapacitance, which greatly enhances the supercapacitive performance. Fe_2_O_3_/N-PC showed a superior capacitance of 290.3 F g^−1^ at 1 A g^−1^ with 93.1% capacity retention after 10,000 charge–discharge cycles. Eventually, a high energy density of 37.6 Wh kg^−1^ at a power density of 1.6 kW kg^−1^ could be delivered with a solid symmetric device.

## 1. Introduction

A supercapacitor (SC) is an attractive energy storage device that offers exceptional power density, charging and discharging rates, and cycle life [1,2,3,4]. In terms of safety and life cycle, SCs are preferred over rechargeable batteries (e.g., Li-selenium [5], Li-sulfur [6], Zn-ion [7], and ammonium-ion batteries [8]). Typically, high-performance SCs greatly depend on the design of electrode materials. Therefore, to develop a new generation of supercapacitors, it is imperative to design and prepare high-performance electrode materials [9,10].

N-doped porous carbon has demonstrated enormous potential for energy storage applications in recent years due to its extraordinary conductivity, large active surface area, and adjustable N-doping [11,12]. However, the N-doped carbon framework suffers from a lower capacity compared with the current anodes of SCs, thus leading to an unsatisfactory energy density of SCs [13,14]. An efficient strategy is to hybridize high-capacity metal oxides with conductive porous carbon to solve the above issue [15,16].

Fe_2_O_3_ has been deemed an ideal electrode material for SCs due to its abundant resources, environmental benignity, and intrinsic pseudocapacitive properties [17,18,19]. Additionally, the Fe_2_O_3_ nanostructure facilitates electrochemical reactions and enlarges the electrochemical capacity contribution ratio by releasing the volume change [20,21]. Previous results [22,23] suggest that the expected electrochemical performance cannot be simply obtained with the physical mixing of Fe_2_O_3_ and carbon. On one hand, Fe_2_O_3_ wrapped into the surface of a carbon material may exfoliate and lower the capacity. On the other hand, agglomerating Fe_2_O_3_ nanoparticles may result in irreversible electrochemical effects because of the decreasing number of active sites and approaching obstacles between the active site and electrolyte. Encapsulating Fe_2_O_3_ nanoparticles in N-doped carbon is considered one of the most promising approaches to mitigate the aforementioned conflicts. The intrinsic porous structure, high electrical conductivity, and superb wettability induced by nitrogen doping would enable fast electron transport and ion diffusion within the peculiar structure, resulting in excellent electrochemical performance. Liu et al. [24] synthesized Fe_2_O_3_ nanodots deposited onto the surface of nitrogen-doped graphene sheets (Fe_2_O_3_ NDs@NG) using a simple thermal method. NG broadened the ion transport path, reduced ion transport resistance, and buffered the volume expansion caused by Fe_2_O_3_ NDs during the charging–discharging process. Moreover, Fe_2_O_3_ NDs offered a large number of active sites. Benefiting from the synergistic effect between NG and Fe_2_O_3_ NDs, the as-prepared Fe_2_O_3_ NDs@NG exhibited a high specific capacitance of 274 F g^−1^ at 1 A g^−1^ with an enhanced rate capability of 51.1%, remaining at 50 A g^−1^.

Hemin (ferric chloride heme), which contains a Fe-N_4_ chelate structure and two carboxyl groups, is a potential doping agent [25]. In this work, Fe_2_O_3_/N-PC was obtained using direct pyrolysis with Hemin/AC precursors in a N_2_ atmosphere. Ultra-small Fe_2_O_3_ with no visible particle clusters were transformed by precisely controlling the calcination temperature of the Hemin. Pyrolytic AC provided an interconnected porous conductive network, which effectively decreased the charge transfer resistance. A synergistic effect between the superior pseudo-capacitive of Fe_2_O_3_ and the conductivity of N-doped AC resulted in an excellent behavior of Fe_2_O_3_/N-PC. It showed a specific capacitance of 290.3 F g^−1^ at 1 A g^−1^. The as-prepared symmetric supercapacitor device achieved a reasonable energy density of 37.6 Wh kg^−1^ and a power density of 1600 W kg^−1^.

## 2. Results and Discussion

**Figure 1:**
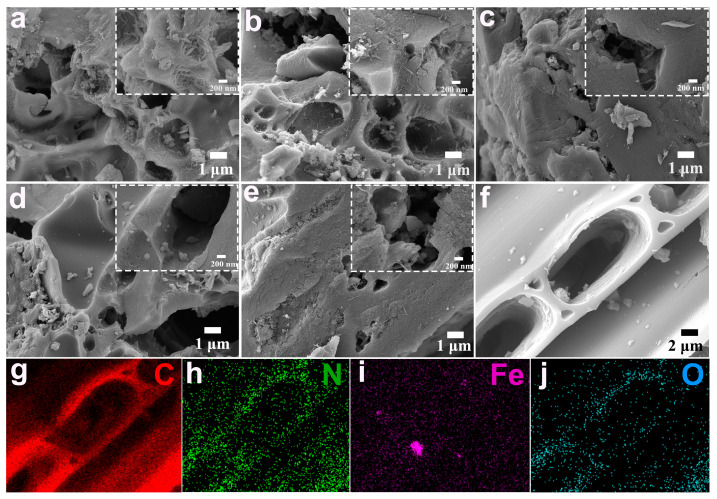
SEM images of (**a**) Hemin/AC, (**b**) Fe_2_O_3_/N-PC 600, (**c**) Fe_2_O_3_/N-PC 700, (**d**) Fe_2_O_3_/N-PC 800, and (**e**) Fe_2_O_3_/N-PC 900. (**f**–**j**) Elemental mapping of Fe_2_O_3_/N-PC 800. The images inside the white dashed line boxes are the corresponding localized magnification.

The morphologies and structures of Hemin/AC and Fe_2_O_3_/N-PC were investigated with SEM, as shown in Figure 1. The 3D architecture could be clearly observed in all the samples. As for Hemin/AC, the Hemin was distributed on the surface of AC (Figure 1a). After heat treatment at 600 °C, AC maintained a porous structure with Hemin crystals still present on the surface of the pore structure (Figure 1b). This was because there was no pronounced decomposition of the Hemin at 600 °C. With an increase in temperature to 700 °C (Figure 1c), few Hemin crystals were present on the surface of the material, which indicated that the Hemin molecule started to decompose. The Hemin crystals were not visible in the SEM images of materials calcined at 800 and 900 °C (Figure 1d,e), which indicated that Hemin was completely decomposed. The samples retained a well-defined nanoporous structure of AC after calcination. This porous structure facilitated the rapid transfer of electrolyte ions during electrochemical processes and provided a conductive substrate for the composite. The elemental mapping images (Figure 1f–j) of Fe_2_O_3_/N-PC ensured the uniform distribution of Fe, N, C, and O on the surface of the electrode.

**Figure 2:**
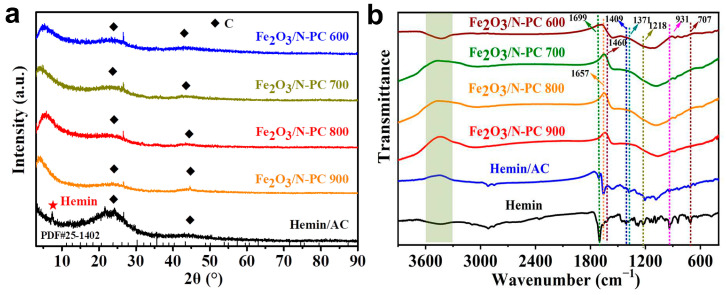
(**a**) XRD patterns. (**b**) FTIR spectra of Hemin/AC, Fe_2_O_3_/N-PC 600, Fe_2_O_3_/N-PC 700, Fe_2_O_3_/N-PC 800, and Fe_2_O_3_/N-PC 900.

The structures of Hemin/AC and Fe_2_O_3_/N-PC were investigated with X-ray diffraction (XRD). As shown in Figure 2a, Hemin/AC exhibited typical diffraction patterns of a discordered carbon structure with a small broad peak at approximately 2θ = 26°and 43°, which corresponded to the (002) and (101) crystal planes of graphitic carbon [26], respectively. A small diffraction peak was detected at 7.5°, attributed to the characteristic diffraction peak of Hemin. This also indicated the successful nanocomposite creation between AC and Hemin. After the pyrolysis reaction, two broad peaks were also detected, which were the characteristic diffractions of amorphous carbon. The weak characteristic peaks at 33.9°, 35.7°, 43.3°, and 63° were attributed to the (109), (119), (0012), and (4012) planes of Fe_2_O_3_ (JCPDS NO.25-1402) [27]. These peaks overlapped with the peaks that corresponded to the amorphous carbon.

The FT-IR spectra of the samples are displayed in Figure 2b. All samples exhibited wide peaks from 3400 to 3500 cm^−1^ (shaded regions), caused by the stretching vibrations of O=C–OH and –OH [28]. The IR spectrum of Hemin/AC retained the characteristic peaks of Hemin, indicating that the molecular structure of Hemin was intact. The peak at 1699 cm^−1^ of Hemin and Hemin/AC was characteristic of its C=O stretching vibration in –COOH [29]. The peak located at 1657 cm^−1^ was derived from the C=C stretching vibration in the porphyrin ring or the C=N stretching vibration in the pyrrole ring [30]. The N–H in-plane vibrations appeared at 1460 cm^−1^. The difference in the IR spectrum before and after the pyrolysis reaction was attributed to the thermal decomposition of Hemin during carbonization. Carbonyls, carboxyls, methoxyls, and other groups might have also detached and condensed [31]. Notably, an absorption peak at 1080 cm^−1^ was found in Fe_2_O_3_/N-PC, which was attributed to the stretching vibration of C-N [32], confirming the successful doping of N into the carbon material.

**Figure 3:**
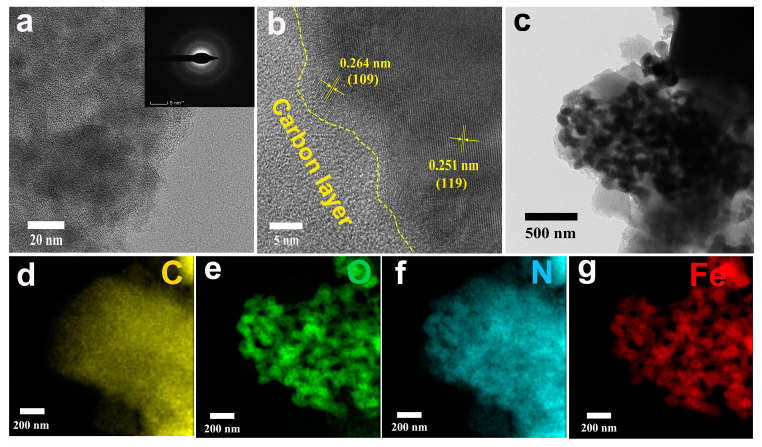
(**a**–**c**) TEM images of Fe_2_O_3_/N-PC 800. (**d**–**g**) Element mapping images of C, O, N, and Fe.

The TEM images (Figure 3) revealed that uniformly dispersed ultrafine Fe_2_O_3_ nanocrystals were embedded in a dense carbon layer. The observed crystallite size of Fe_2_O_3_ was around 10 nm due to the spatial nanoconfinement effect of porous activated carbon (Figure 3a). Ultrafine Fe_2_O_3_ nanocrystals presented a large specific surface area, which effectively promoted the electrode/electrolyte contact area and sufficient accessible active sites. This enabled a fast-transferring channel for electrolyte ions and electrons. High-resolution TEM (HRTEM) analysis showed that Fe_2_O_3_/N-PC possesses a nanonetwork in which Fe_2_O_3_ nanocrystals are wrapped by carbon. The HRTEM image (Figure 3b) showed clear and ordered lattice fringes with a lattice spacing of 0.264 and 0.251 nm, corresponding to the (109) and (119) planes of Fe_2_O_3_. Twin boundaries (depicted by yellow lines) were prominent, suggesting the formation of interfaces. The Fe_2_O_3_/N-PC interface further promoted charge storage on the surface or near the surface of the electrode and thus efficiently hindered the volume change in Fe_2_O_3_ during the charge/discharge process. These changes were expected to enhance the cycling stability of Fe_2_O_3_/N-PC.

**Figure 4:**
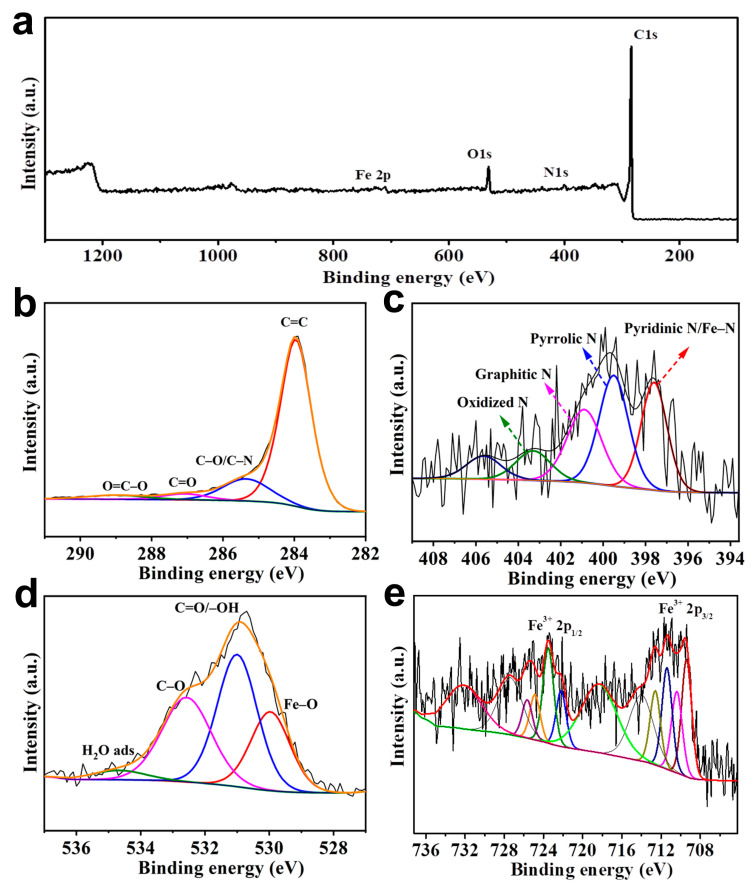
(**a**) XPS survey spectra of Fe_2_O_3_/N-PC. (**b**–**e**) C 1s, N 1s, O 1s, and Fe 2p spectra of Fe_2_O_3_/N-PC.

The chemical composition of Fe_2_O_3_/N-PC was analyzed with X-ray photoelectron spectroscopy (XPS) (Figure 4). As shown in Figure 4a, the obtained peaks of Fe 2p, C 1s, N 1s, and O 1s indicated that Fe_2_O_3_ was successfully embedded in the carbon layer. The C 1s spectrum was fitted into four subpeaks, including C=O, C–O/C–N, C=O, and O=C–O at 283.9, 285.5, 287.1, and 288.9 eV [33], respectively (Figure 4b). In addition, the N 1s spectrum (Figure 4c) could be fitted into four subpeaks situated at 397.6, 399.5, 400.9, and 403.2 eV, corresponding to pyridine N, pyrrolic N, graphitic N, and oxidized N [34], respectively. Pyrrolic N can improve the wettability of carbon materials in an aqueous electrolyte. Pyridine N can be attributed to sp2 hybridization, which can improve the conductivity of electrons, thus enabling rapid electron transfer. In Figure 4d, three peaks at 529.9 eV (Fe–O), 531.1 eV (C=O/–OH), and 532.6 eV (C–O) [35] were identified in the O 1s spectrum. Two major peaks of Fe 2p (Figure 4e) were detected at 711.2 and 724.6 eV, attributed to the Fe 2p_3/2_ and Fe 2p_1/2_ peaks of Fe_2_O_3_, respectively [36]. Additionally, a satellite peak at 719.5 eV showed the existence of Fe^3+^ [37]. The element compositions (atomic %) of different samples determined with XPS are listed in Table 1. The XPS analysis showed that the percentage of the overall N and Fe showed an increase followed by a gradual decrease with increasing pyrolysis temperature. The highest content of N and Fe was observed in Fe_2_O_3_/N-PC 800. A higher content of N resulted in better electronic conductivity. A higher content of Fe contributed to pseudocapacitance. These factors played a key role in improving the electrochemical performance of electrode materials. Therefore, Fe_2_O_3_/N-PC 800 might achieve excellent electrochemical performance.

**Figure 5:**
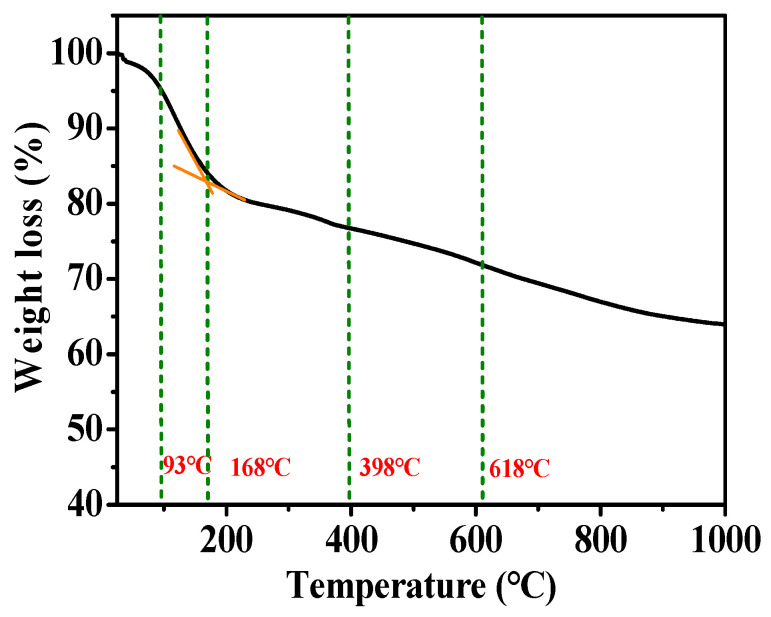
TG curves of Hemin/AC.

The thermal stability of Hemin/AC was evaluated with TG analysis (Figure 5). Weight loss below 93 °C corresponded to the loss of crystalline water and surface adsorbed water in Hemin/AC. Weight loss between 93 and 168 °C corresponded to the removal of adsorbed water in the porous structure of Hemin/AC. Weight loss between 168 and 398 °C corresponded to the removal of Hemin axial chloride [38], and weight loss between 398 and 689 °C corresponded to the decomposition of organic groups outside the porphyrin ring of the Hemin molecule, such as –CH_2_CH_2_COOH, –CH_3_, and –CH=CH_2_. The decomposition of the porphyrin ring in the center of the Hemin molecule occurred at 689–900 °C [38,39].

**Figure 6:**
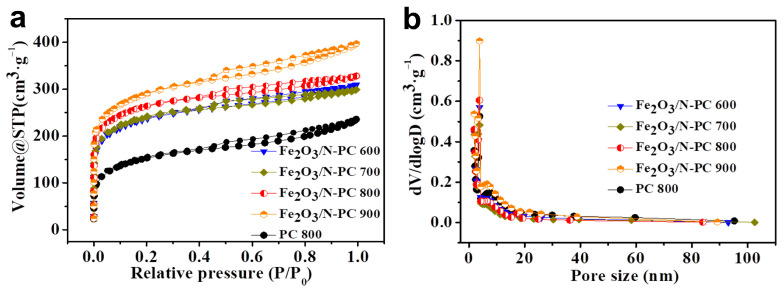
(**a**) Nitrogen adsorption–desorption isotherms. (**b**) Pore size distribution.

The N_2_ adsorption/desorption isotherms and pore size distribution of the obtained materials are presented in Figure 6a. The isotherm of PC and Fe_2_O_3_/N-PC displayed a type IV isotherm and an H4-type hysteresis loop, implying a mesoporous structure [40]. The surface area, pore volume, and pore size of the sample are presented in Table 2. The Fe_2_O_3_/N-PC 800 had an average BET-specific surface area of 953.3 m^2^ g^−1^, a mean pore size of 2.1 nm, and an average BJH-specific pore volume of 0.51 cm^3^ g^−1^. The micropore surface area was 614.5 m^2^ g^−1^ (pore volume of 0.26 cm^3^  g^−1^), of which the micropore contribution rate was 51%. The results confirmed that the calcination temperature and the addition of Hemin strongly affected the structure of the materials. The carbonization procedure greatly increased the specific surface area. The BJH pore size distribution curves (Figure 6b) indicated that micropores and mesopores were relatively concentrated, and the proportion of micropores was higher. Mesopores provided low-resistance channels for electrolyte ions to enter the internal pores. As a storage place for electrolyte ions, micro-pores provided effective adsorption sites. Furthermore, the introduction of surface heteroatoms (N, O) into the carbon skeleton often changes the electrical conductivity, wettability, and capacitance performance of carbon materials. The synergistic effect of these features substantially improved the electrochemical performance of the Fe_2_O_3_/N-PC 800 electrode. 

**Figure 7:**
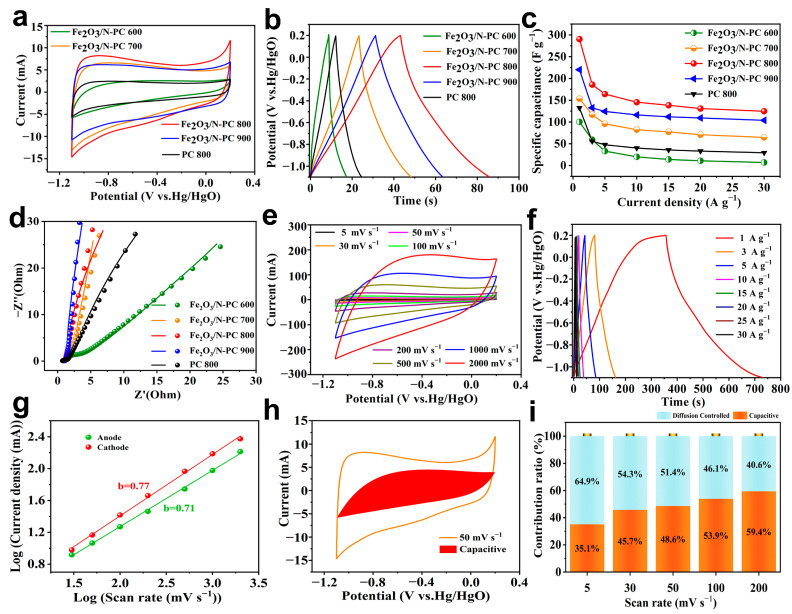
(**a**) CV curves at 50 mV s^−1^. (**b**) GCD curves at 5 A g^−1^. (**c**) Specific capacitance at different current densities. (**d**) EIS spectra and the corresponding fitting curves of various electrodes. (**e**) CV curves at scan rates from 1 to 2000 mV s^−1^. (**f**) GCD curves at current densities from 1 to 30 A g^−1^. (**g**) The relationship between peak current and scan rate. (**h**) Capacitive and diffusion-controlled charge storage contributions at a scan rate of 50 mV s^−1^. (**i**) Capacitive contributions for Fe_2_O_3_/N-PC electrode at different scan rates.

The electrochemical performance of the samples was evaluated using a common three-electrode system using 6 M KOH as the electrolyte. Figure 7a illustrates the CV curves of different samples at a scan rate of 50 mV s^−1^ within the potential of −1.1–0.2 V. Due to the combination of electrical double layer capacitance (EDLC) and pseudocapacitance, all profiles displayed a quasi-rectangular shape with a non-negligible peak in low-potential regions. The conductive carbon layer produced the EDLC. Pseudocapacitance was probably caused by redox reactions involving Fe_2_O_3_ in the electrode surface and by the following reactions [41]:

Fe^II^O + 2OH^−^ ↔ Fe^II^(OH)_2_ + 2e^−^

2Fe^II^O + 2OH^−^ ↔ (Fe^III^O)^+^(OH)_2_ (Fe^m^O)^+^ + 2e^−^

In contrast to other materials, Fe_2_O_3_/N-PC 800 had a slightly larger CV area, which indicated a higher specific capacity. The approximately symmetrical triangular shape of the GCD curves at a current density of 5 A g^−1^ (Figure 7b) showed the EDLC and pseudocapacitor charge-storage mechanisms, confirming the CV results [42]. The specific capacitance of Fe_2_O_3_/N-PC 800 calculated at a current density of 5 A g^−1^ was 164.2 F g^−1^, which was higher than the specific capacitance of Fe_2_O_3_/N-PC 600 (33.1 F g^−1^), Fe_2_O_3_/N-PC 700 (95.8 F g^−1^), Fe_2_O_3_/N-PC 900 (124.2 F g^−1^), and PC 800 (47.7 F g^−1^). The gravitational specific capacitance of different electrode materials at different current densities was calculated based on the GCD curves (Figure 7c). The Fe_2_O_3_/N-PC 800 electrode exhibited the best charge storage capacity. A computational fit of EIS impedance spectroscopy of PC and Fe_2_O_3_/N-PC electrodes is shown in Figure 7d. As shown in the figure, the results of fitting relations and experimental data did not differ substantially. The Nyquist plot consisted of a semicircle at high frequency, followed by a slope line in the low-frequency range. This could be blamed on charge transfer and ions diffusion kinetics at the electrode/electrolyte interface [43]. The R_ct_ of Fe_2_O_3_/N-PC 800 was below the other electrodes, indicating that the charge transfer that occurred in electrode/electrolyte interphase is monitored by the well-defined nanoporous structure. The straight line in the low-frequency region indicated the superior ion transport capability and frequency responsiveness of Fe_2_O_3_/N-PC 800 [44]. Due to the fast ion response, the Fe_2_O_3_/N-PC 800 CV curves (Figure 7e) retained a quasi-rectangular shape up to 2000 mV s^−1^. The GCD curves of Fe_2_O_3_/N-PC 800 at various current densities exhibited almost symmetrical triangular shapes, indicating excellent electrochemical reversibility and capacitive behavior (Figure 7f). The Fe_2_O_3_/N-PC 800 electrode showed specific capacitance values of 290.3, 185.8, 164.2, 145.4, 138.5, 130.8, and 124.6 F g^−1^ at current densities of 1, 3, 5, 10, 15, 20, and 30 A g^−1^, respectively.

Additionally, the charge storage dynamics diffusion process was performed based on the CV curves at different scanning rates using the following equations [45]:

i_p_ = aυ^b^ (1)

i(V) = k_1_υ + k_2_υ^1/2^ (2)

where i is the current (A), υ is the current (V s^−1^), a and b are the adjustable values, respectively. In general, b = 0.5 indicates a diffusion-controlled process, whereas b = 1 indicates a capacitance-controlled process [46]. Based on the CV profiles and linear fitting lines of logυ vs. logi (Figure 7g), the b values of cathodic and anodic peaks were found to be 0.77 and 0.71, respectively, i.e., close to 1. This showed that the kinetics of the Fe_2_O_3_/N-PC 800 was mainly dominated by a capacitance-controlled behavior. The CV curve at 50 mV s^−1^ and the percentage of capacitance contribution of the Fe_2_O_3_/N-PC 800 electrode are shown in Figure 7h. The capacitance contribution at different scan rates is illustrated in Figure 7i. The contribution of pseudocapacitance behavior for the Fe_2_O_3_/N-PC 800 electrode gradually increased as the scan rate increased. The percentage contribution of capacitive control for the Fe_2_O_3_/N-PC 800 electrode was 35.1%, 45.7%, 48.6%, 53.9%, and 59.4% at scan rates of 5 mV s^−1^, 30 mV s^−1^, 50 mV s^−1^, 100 mV s^−1^, and 200 mV s^−1^, respectively.

These results indicated the outstanding electrochemical performance of the Fe_2_O_3_/N-PC 800 electrode, which might be attributed to several aspects. First, the reasonable pore structure distribution, large specific surface area, and the presence of N-doping provided the Fe_2_O_3_/N-PC 800 electrode with more charge storage and transfer space, as well as better wettability in KOH electrolytes. Second, the three-dimensional conductive network around each Fe_2_O_3_ nanoparticle offered many accessible active sites and a highly interconnected porous conductive network, which enabled easy diffusion and permeation of the electrolyte and thus efficiently improved the reaction rate. Furthermore, the ultra-small Fe_2_O_3_ particles were uniformly and densely distributed on N-PC, alleviating the pulverization and aggregation caused by the volume change and simultaneously boosting their utilization efficiency during pseudocapacitive reactions. 

**Figure 8:**
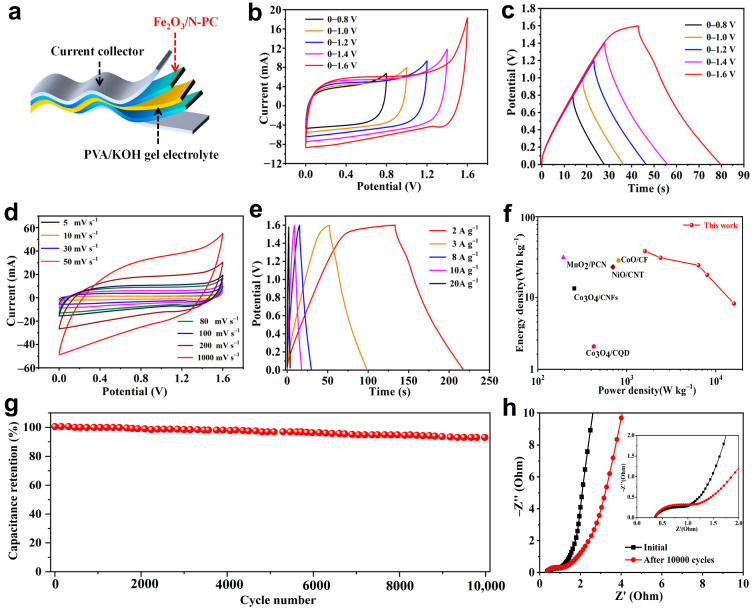
The electrochemical performance of the quasi-solid symmetrical Fe_2_O_3_/N-PC 800 device. (**a**) The structural model of the quasi-solid-state supercapacitor. (**b**) CV curves at 50 mV s^−1^ from 1–1.6 V. (**c**) GCD curves at 5 A g^−1^ from 1–1.6 V. (**d**) CV curves at various scan rates. (**e**) GCD curves under various current densities. (**f**) Ragone plot of the symmetric supercapacitor. (**g**) Cycling stability of Fe_2_O_3_/N-PC 800 for 10,000 cycles measured at 10 A g^−1^. (**h**) Nyquist plots of the symmetric supercapacitor.

To further characterize the electrochemical characteristics of Fe_2_O_3_/N-PC 800, a symmetrical supercapacitor (Figure 8a) was assembled in the PVA/KOH sol electrolyte. The Fe_2_O_3_/N-PC 800//Fe_2_O_3_/N-PC 800 symmetrical supercapacitor could be stably performed in the voltage range of 0–1.6 V (Figure 8b,c). Figure 8d shows the CV curves of the symmetrical supercapacitor at different scan rates. Even at a high scanning rate of 1000 mV s^−1^, its rectangular shape was maintained, which indicated excellent current response capability. The GCD curves (Figure 8e) had a nonsymmetrical shape and a negligible voltage drop, which indicated a good capacitive behavior, contributed to a pseudocapacitive behavior, and suggested good Coulombic efficiency. According to the GCD curves, the symmetrical supercapacitor displayed a large specific capacitance of 105.8 F g^−1^ at 2 A g^−1^. The Ragone diagram is shown in Figure 8f. The energy density of the Fe_2_O_3_/N-PC 800-based symmetric supercapacitor was 37.6 Wh kg^−1^ at a power density of 1600 W kg^−1^. The high energy and power densities of Fe_2_O_3_/N-PC 800 exhibited better performance than those of recently reported similar SCs, such as MnO_2_/PCN (31.3 Wh kg^−1^ at 193.6 W kg^−1^) [47], Co_3_O_4_/CNFs (13 Wh kg^−1^ at 257 W kg^−1^) [48], NiO/CNT (23.9 Wh kg^−1^ at 698.6 W kg^−1^) [49], CoO/CF (28.8 Wh kg^−1^ at 804.3 W kg^−1^) [50], and Co_3_O_4_/CQD (2.49 Wh kg^−1^ at 426 W kg^−1^) [51] and also shown in the Ragone plot. The symmetric supercapacitor showed outstanding cycle stability after 10,000 cycles at 10 A g^−1^, and the capacitance retention was 93.1% (Figure 8g). The impedance data before and after cycling are shown in Figure 8h. The EIS curves after 10,000 cycles showed a minor effect on the high-frequency range and a decrease in the slope of the low-frequency data. These changes also showed the excellent electrochemical stability of the device.

## 3. Experimental Section

### 3.1. Preparation of Fe_2_O_3_ Embedded in N-Doped Porous Carbon (Fe_2_O_3_/N-PC) 

**Figure 9:**
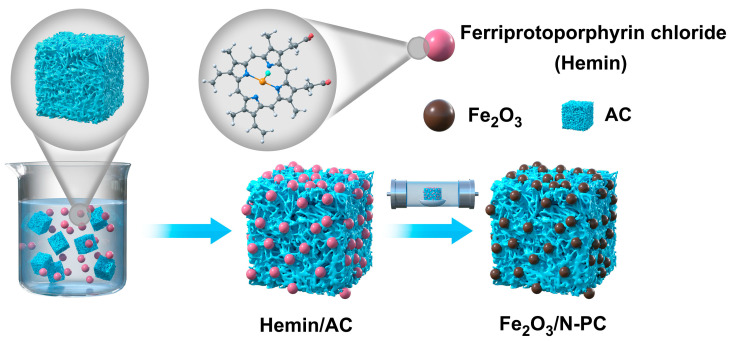
Schematic diagram of Fe_2_O_3_/N-PC synthesis.

Hemin (95%) and AC were purchased from Aladdin (Shanghai, China). Fe_2_O_3_/N-PC was prepared following a typical procedure, as illustrated in Figure 9. Firstly, Hemin and activated carbon were mixed in 40 mL of N,N-Dimethylformamide (DMF) at a mass ratio of 1:1. Then, it was sonicated for 30 min to form a Hemin/AC suspension. After high-speed centrifugation, the product was collected, washed with ethanol, and then dried for 12 h at 60 °C. Subsequently, 5 g of the sample was annealed at 800 °C for 3 h at a heating rate of 5 °C min^−1^ under N_2_ atmosphere (40 mL min^−1^ gas flow rate). Finally, the collected material was washed with distilled water to neutrality and dried at 90 °C, the product was named Fe_2_O_3_/N-PC 800. For comparison, Fe_2_O_3_/N-PC 600, Fe_2_O_3_/N-PC 700, and Fe_2_O_3_/N-PC 900 (temperatures were 600, 700, and 900 °C, respectively) were also prepared following the same procedure. The product obtained by calcinating AC at 800 °C without Hemin was noted as PC 800.

### 3.2. Preparation of the Fe_2_O_3_/N-PC Electrode

Fe_2_O_3_/N-PC, Polytetrafluoroethylene (PTFE), and acetylene black were dispersed in ethanol to prepare a homogeneous black slurry at a mass ratio of 8:1:1. Then, the slurry was coated on nickel foam (1 × 1 cm^2^) and dried at 60 °C for 12 h. The prepared electrode was compressed under 8 MPa. The mass loading of the Fe_2_O_3_/N-PC on the individual electrode was approximately 1.5 mg.

### 3.3. Fabrication of Symmetric SCs

A symmetric SC was fabricated by wrapping the PVA-KOH gel electrolyte with two Fe_2_O_3_/N-PCelectrodes. The method of PVA-KOH gel electrolyte for the Fe_2_O_3_/N-PC supercapacitor was as follows: 10 mL of deionized water was added with 6 g KOH and 60 mL of deionized water was added with 6 g PVA. To obtain a semitransparent PVA/KOH gel electrolyte, the KOH solution was dropwise mixed with PVA solution at 90 °C with slow stirring. The symmetric Fe_2_O_3_/N-PC electrodes were coated with the resultant gel electrolyte before the supercapacitor was assembled. Then, the two electrodes were overlaid head-to-head. The uncoated part of the nickel foam served as the conductive electrode ear of the device. Finally, the device was obtained after being packaged with polyethylene film.

### 3.4. Characterization

The morphologies of the as-prepared samples were characterized using a Hitachi S-4800 field-emission scanning electron microscope (FE-SEM) (Hitachi, Ltd., Tokyo, Japan) with an energy dispersive X-ray spectrometer (EDX). X-ray diffraction (XRD) was performed on a Bruker D8 DISCOVER X-ray diffractometer (Bruker, Karlsruhe, Germany) equipped with Cu Kα radiation (λ = 0.15406 nm). Fourier-transform infrared (FT-IR) spectra were recorded at room temperature using a Nicolet-20DXB instrument (Thermo Scientific, Waltham, MA, USA). The samples were further observed with an FEI Talos F200XG2 AEMC transmission electron microscopy (TEM) (FEI Company, Hillsborough, NC, USA). X-ray photoelectron spectroscopy (XPS) spectra were measured with a Thermo Scientific K-Alpha spectrometer (Thermo Fisher Scientific, Waltham, MA, USA) with a monochromatized Al Kα X-ray source (1486.6 eV). Thermogravimetric analysis (TGA) was performed on a Rigaku TG-DTA8122 (Rigaku, Tokyo, Japan). N_2_ adsorption–desorption isotherms were recorded with Brunauer–Emmett–Teller (BET) using an ASAP2460 (Micromeritics, Atlanta, GA, USA) under liquid nitrogen temperature (77 K). 

### 3.5. Electrochemical Characterization

A three-electrode system with 6 M KOH solution was prepared to measure the electrochemical performance of the Fe_2_O_3_/N-PC electrode, and a platinum foil and Hg/HgO electrode acted as the counter electrode and reference electrode, respectively. The electrochemical characteristics were evaluated using a CHI 760E electrochemical workstation (Chenhua, Shanghai, China), including cyclic voltammetry (CV), galvanostatic charge/discharge (GCD) measurements, and electrochemical impedance spectroscopy (EIS).

The capacitance (*C_s_*, F g^−1^) of the electrode was evaluated from the GCD curve according to the following equations.

For a three-electrode configuration:

(3) $$C=\frac{I\Delta t}{m\Delta U}$$

where *C* (F g^−1^) is the gravimetric specific capacitance of the working electrode; *I* (A) represents the discharge current; *t* (s) stands for discharge time; ∆*U* (V) is voltage range excluding the IR drop; and *m* (g) is the mass loading of active material in a single electrode.

For a two-electrode configuration:

(4) $$C_{s}=4\frac{I\Delta t}{M\Delta U}$$

where *C_s_* (F g^−1^) is the area-specific capacitances of the working electrode; *I* (A) represents the discharge current; *t* (s) stands for discharge time; ∆*U* (V) is the voltage range excluding the IR drop; and *M* (g) is the total mass of active material. 

The energy density and power density of a symmetric supercapacitor can be calculated using the following equations:

(5) $$E=\frac{0.5\Delta U^{2}}{3.6}(\frac{C_{s}}{4})$$

(6) $$P=\frac{3600E}{\Delta t}$$

The device’s energy density is represented by *E* (Wh kg^−1^) and power density by *P* (W kg^−1^).

## 4. Conclusions

In summary, N-doped porous carbon embedded with ultra-small Fe_2_O_3_ (Fe_2_O_3_/N-PC) was fabricated with electrostatic adsorption and subsequent heat treatment. The N-doped porous carbon provided a continuous conductive network in the Fe_2_O_3_/N-PC. Meanwhile, Fe_2_O_3_ provided sufficient active sites, which facilitated fast ion diffusion and electron transfer at the electrode/electrolyte interface. The strong synergistic contribution of pseudo-capacitive Fe_2_O_3_ and N-PC improved the electrochemical properties of Fe_2_O_3_/N-PC. As a consequence, the Fe_2_O_3_/N-PC sample exhibited a high specific capacitance of 290.3 F g^−1^ at 1 A g^−1^. The assembled Fe_2_O_3_/N-PC 800//Fe_2_O_3_/N-PC 800 symmetrical supercapacitor operated at a broad potential range of 1.6 V and delivered a high capacitance of 105.8 F g^−1^ at 2 A g^−1^ with a stable cycling life (93%) over 10,000 cycles. In addition, the energy density of the Fe_2_O_3_/N-PC 800-based symmetric solid-state supercapacitor was 37.6 Wh kg^−1^ at a power density of 1600 W kg^−1^, which exceeded the power density of materials recently reported in a similar study. Overall, in this study, we provided a simple design strategy for developing new electrode materials in high-performance SCs.

## Figures and Tables

**Table 1:** The content of C, N, O, and Fe elements in different samples.

Sample	C (at%)	N (at%)	O (at%)	Fe (at%)
PC 800	91.44	/	8.56	/
Fe_2_O_3_/N-PC 600	83.33	2.95	12.85	0.87
Fe_2_O_3_/N-PC 700	85.09	3.12	10.88	0.91
Fe_2_O_3_/N-PC 800	87.56	3.56	7.84	1.04
Fe_2_O_3_/N-PC 900	90.46	1.92	7.23	0.39

**Table 2:** Pore structure characteristics of PC 800, Fe_2_O_3_/N-PC 600, Fe_2_O_3_/N-PC 700, Fe_2_O_3_/N-PC 800, and Fe_2_O_3_/N-PC 900.

Sample	S_BET_ (m^2^ g^−1^)	V_BET_ (cm^3^ g^−1^)	S_BET-micro_ (m^2^ g^−1^)	V_BET-micro_ (cm^3^ g^−1^)	Dave (nm)
PC 800	539.9	0.36	274.0	0.12	2.7
Fe_2_O_3_/N-PC 600	852.7	0.48	542.8	0.23	2.3
Fe_2_O_3_/N-PC 700	866.2	0.46	571.4	0.24	2.1
Fe_2_O_3_/N-PC 800	953.3	0.51	614.5	0.26	2.1
Fe_2_O_3_/N-PC 900	1044.5	0.61	673.5	0.28	2.4

## Data Availability

The data are contained within this article.
